# annoreport: an interactive tool for metagenome annotation

**DOI:** 10.1093/bioadv/vbag257

**Published:** 2026-09-02

**Authors:** Kepler Ridge, Byron J Adams

**Affiliations:** Department of Biology, Brigham Young University, Provo, UT, 84602, United States; Department of Biology, Brigham Young University, Provo, UT, 84602, United States

## Abstract

**Summary:**

Gene annotation of metagenome-assembled genomes is a critical step in determining the functional potential of microbial communities from environmental samples. However, annotation workflows using tools such as Prokka or Bakta produce per-bin output with 10 to 14 files per bin, making manual review infeasible at scale. Existing tools incompletely aggregate and visualize gene annotation content across an entire metagenomic dataset. Here we present annoreport, a single-script Python tool requiring no external dependencies beyond Python 3.9+ that accepts output from either Prokka or Bakta, automatically detecting the annotation tool used. annoreport produces an interactive web-based report summarizing gene product frequencies, hypothetical protein rates, feature type distributions, and functional gene clustering via UniProt annotation across all bins. Applied to 206 metagenome-assembled genomes from Antarctic soil metagenomes, annoreport identified 603,799 coding sequences with a 47.1% annotation rate and revealed functional categorization in Transport & Membrane, Nucleotide Binding, and DNA Metabolism categories.

**Availability and implementation:**

Freely available at https://github.com/keplerridge/annoreport under MIT license, via Bioconda (annoreport) and PyPI (annoreport).

## 1 Introduction

Metagenome-assembled genomes (MAGs) have become foundational to microbial ecology and allow for functional inference across diverse environments (Mirete *et al.* 2025). MAG-based studies increasingly attempt to not only recover genomes, but also to infer the functional architecture of entire microbial communities across environmental gradients, disturbance regimes, and ecosystem states (Krause *et al.* 2014). As sequencing and binning workflows now routinely recover hundreds to thousands of MAGs from a single study, interpretation increasingly depends on the ability to synthesize annotation content across genomes into biologically interpretable patterns. Patterns in gene content can reveal dominant metabolic strategies, signatures of environmental filtering, stress-response capacity, nutrient acquisition strategies, and the prevalence of novel or poorly characterized functions within a system (Dragone *et al.* 2022, Thompson *et al.* 2025). This challenge is particularly relevant in extreme or under-characterized environments, where large fractions of coding sequences often remain functionally unclassified. Despite the growing importance of community-scale functional interpretation in microbial ecology, existing annotation workflows largely remain organized around per-bin outputs rather than ecosystem-level synthesis. By aggregating patterns across large MAG collections, annoreport supports emerging trait-based approaches in microbial ecology, where distributions of functional capabilities across communities can be used to infer ecological strategies, environmental filtering, and community functional structure. Such a community-scale synthesis and framework enables rapid exploration of ecosystem-level trait distributions from genome-resolved metagenomic datasets.

As MAG-based workflows have scaled to recover hundreds of bins from a single sequencing project, the need for rapid, interpretable summaries of annotation results has also grown. Tools like Prokka (Seemann 2014) and Bakta (Schwengers *et al.* 2021) have become standard for prokaryotic genome annotation, producing per-bin output files containing gene product names, feature types, Enzyme Commission numbers, and clusters of orthologous groups. While both tools provide invaluable information, neither one provides a cross-bin summary layer which leaves researchers to manually aggregate results across anywhere from dozens to hundreds of output files to assess overall functional content, annotation rates, or the distribution of hypothetical proteins.

Existing tools partially address these problems, but gaps remain. MetaErg (Dong and Strous 2019) provides HTML-based annotation visualization but operates as a full re-annotation pipeline rather than a post-processing step for existing Prokka or Bakta output, requiring significantly greater computational resources. MultiQC (Ewels *et al.* 2016) aggregates results across samples into a single interactive report but is designed for sequencing quality control metrics rather than gene annotation content. MAGFlow and BIgMAG (Yepes-García and Falquet 2024) provide interactive dashboards for MAG quality assessment but focus on completeness and contamination metrics rather than functional annotation summaries. While these tools serve as a complement to annotation workflows by addressing quality control and assembly metrics, none summarize gene annotation content directly (Table 1). To our knowledge, no lightweight, dependency-free tool currently exists to summarize and visualize gene annotation content directly from Prokka or Bakta output across an entire MAG dataset.

**Table 1:** Comparison of annoreport with existing tools MAG annotation summarization.

Tool	Accepts Prokka/Bakta output	Zero dependencies	UniProt annotation	Functional clustering
**annoreport**	✓	✓	✓	✓
**MetaErg**	✗	✗	✗	Partial
**MultiQC**	Partial	✗	✗	✗
**MAGFlow/BIgMAG**	✗	✗	✗	✗

MAGs, metagenome-assembled genomes. ✓, feature present. ✗, feature absent.

## 2 Implementation

annoreport is implemented as a single Python script using only the standard library, requiring no installation beyond Python 3.9+. annoreport automatically detects whether annotation was done with Bakta or Prokka, simplifying integration into existing workflows. Tool type can also be declared explicitly with the --tool flag. UniProt annotation lookup is enabled by default. The queries to UniProt are rate-limited at one every 0.2 seconds to respect UniProt’s resources. In offline mode, or for a quicker report, users can use the --no_uniprot flag to turn this feature off. This will exclude the gene function clustering as well as the gene name and function columns in the summary table. One of the key features of annoreport is the functional gene clustering. Genes are clustered into one of ten biological categories (DNA Metabolism, Transcription, Translation & Ribosomes, Energy & Metabolism, Transport & Membrane, Stress & Chaperones, Cell Division & Structure, Signaling & Regulation, Nucleotide Binding, and Other/Unclassified) using a priority-ordered keyword matching scheme applied to the UniProt annotations. This categorization framework was designed to provide an interpretable ecological summary rather than a formal ontology-based annotation system, enabling researchers to rapidly identify broad patterns in community functional composition without replacing pathway reconstruction or curated metabolic inference pipelines. Accordingly, categories were selected to capture broad functional themes commonly used in microbial ecological and environmental genomics, broadly mirroring clusters of orthologous groups (COG) produced by Prokka and KEGG metabolic pathways (Kanehisa *et al.* 2023), providing a familiar functional framework. As category keywords are drawn directly from UniProt’s controlled vocabulary, a manually curated, peer-reviewed annotation resource, assignments inherit the biological validity of UniProt curation. annoreport is designed to provide interpretable ecological summaries rather than formal functional predictions, and category assignments should be interpreted as broad functional themes consistent with UniProt-curated annotations rather than statistically validated classifications. The output from annoreport is an interactive HTML file, with no JavaScript dependencies, and a summary TSV for downstream use in R, Python, or Excel, enabling rapid identification of dominant functional patterns across large MAG collections.

## 3 Features and usage

annoreport is designed for ease of use across computational skill levels. It has a command line interface with five arguments. The only required argument is—annotation_dir and needs the path to the folder holding the annotation results that the users want to summarize. Other optional flags allow users to specify an output directory (--outdir, default: annotation_summary), the number of gene products to summarize (--top_n, default: 100), explicit tool declaration (--tool, default: auto-detect), and offline mode (--no_uniprot, default: off).

The HTML report has several sections to make interpretation of annotation easier. The first section shows summary statistics of the annotation itself. There are cards showing how many bins/MAGs were annotated, the number of contigs and N50, the assembly size, the total coding sequences (CDS), the annotation rate, and how many RNA features there are. A dedicated card shows a count and percentage of hypothetical proteins, which are CDS with no known function that are excluded from the rest of the summary. Below the hypothetical protein callout, there is a summary which breaks down by percentage the distribution and count of different feature types. An adjacent card provides an RNA features breakdown including the top Enzyme Commission numbers (EC), as well as a section with the top COGs, with the associated counts. Because of the way Prokka and Bakta differ in function, the COG summary from a Bakta annotation would likely be very sparse or empty.

The next card on the page shows the functional gene clustering. This card has subcards for each cluster with the cluster name, the number of genes, the number of CDS, and a list of the genes in that cluster with their associated counts. Following the clustering card there is another cluster summary card that shows the distribution of genes in each cluster. The final card is a searchable table of the top N annotated gene products. There is also a TSV produced with the columns of rank (by CDS frequency), count, percent_of_total_cds, product, gene_name, function, and keywords. This TSV is available for downstream analysis in R, Python, or Excel.

## 4 Use case and example

**Figure 1:**
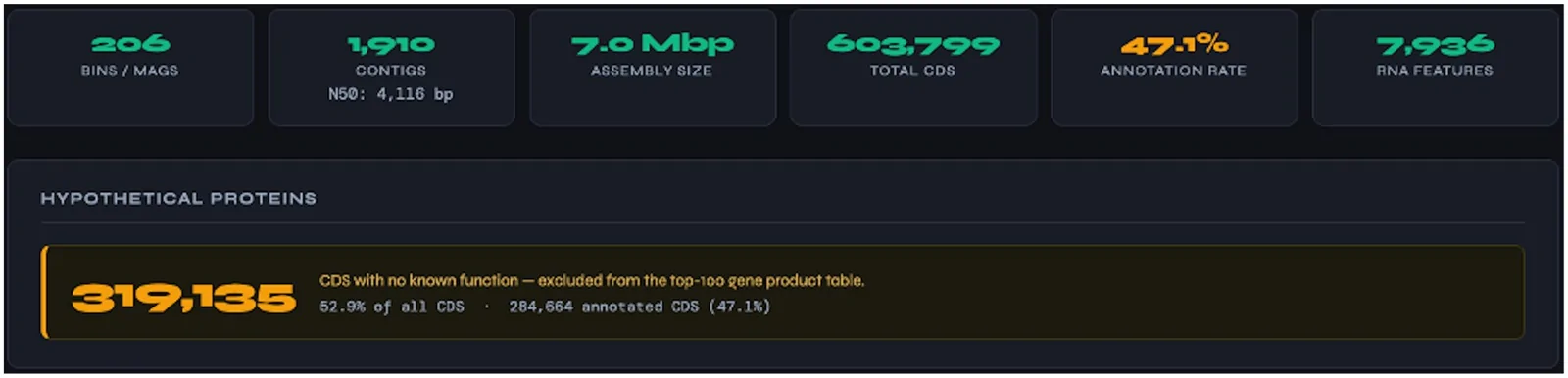
HTML summary report generated by annoreport showing assembly statistics, hypothetical protein callout, and feature type distribution across 206 MAGs annotated with Bakta.

**Figure 2:**
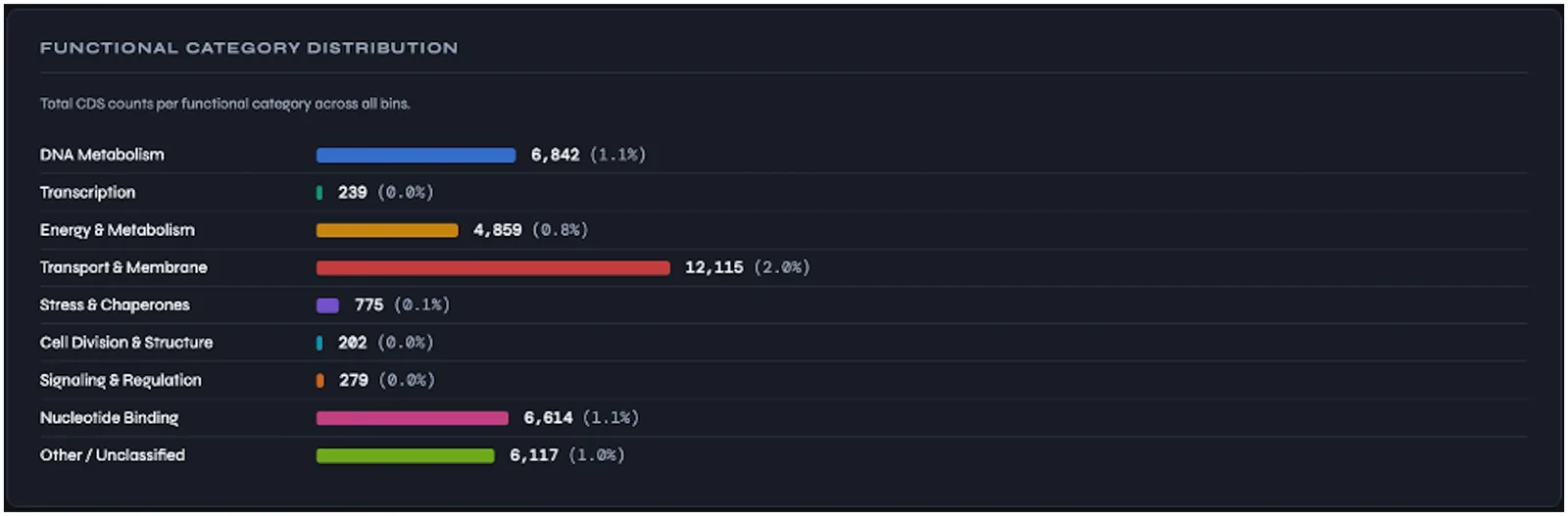
Functional category distribution of the top 100 annotated gene products across 206 MAGs from Antarctic soil, as generated by annoreport from Bakta annotation output. CDS counts are shown per functional category assigned by priority-ordered UniProt keyword matching.

To demonstrate annoreport, we applied it to metagenomic annotations derived from 18 soil samples from the McMurdo Dry Valleys in Antarctica (Thompson *et al.* 2025). Raw paired-end reads were independently quality trimmed using fastp (Chen 2023) and co-assembled using MEGAHIT (Li *et al.* 2015) and binned using MetaBAT2 (Kang *et al.* 2019) for this demonstration. MAGs were subsequently annotated with both Prokka v1.15.6 using the --metagenome flag and with Bakta v1.12.0 using the --meta flag and the light database v6.0, with all other parameters set to default for both tools. annoreport automatically detected the annotation tool and generated a summary report for each. Across the 206 Bakta-annotated MAGs, annoreport identified 603 799 CDS with an annotation rate of 47.1%, of which 52.9% were hypothetical proteins (319 135 CDS), a proportion consistent with the highly novel microbial diversity characteristic of Antarctic soil environments (Thompson *et al.* 2025) (Fig. 1). The large proportion of hypothetical proteins highlights both the extent of unexplored functional diversity in Antarctic soil microbiomes and the importance of tools capable of summarizing annotation uncertainty and novelty at the community scale. RNA parsing identified 6593 tRNA, 103 rRNA, and 83 tmRNA features across the dataset. Functional clustering of the top 100 annotated gene products revealed predominance in Transport & Membrane, Nucleotide Binding, and DNA Metabolism (Fig. 2). annoreport successfully processed both Prokka and Bakta output directories using automatic tool detection, producing an identical report structure for each.

## 5 Performance

annoreport was benchmarked on an Intel Xeon Gold 6230 CPU (2.10 GHz, 40 cores, 752 GB RAM) using a single thread. Results demonstrated that annoreport processed 206 MAGs in 75.7 ± 3.5 s (Prokka) and 76.8 ± 3.7 s (Bakta) with UniProt annotation enabled, and 11.7 ± 0.1 s (Prokka) and 12.5 ± 0.2 s (Bakta) without UniProt annotation. Peak memory usage remained low across all configurations, ranging from 24.6 to 42.4 MB. GFF-only mode, which enables annoreport to process annotation files from public genome catalogues without requiring full tool output, performed comparably to full output mode with a runtime of 79.4 ± 0.4 s with UniProt annotation and 17.0 ± 0.2 s without. To evaluate performance at larger scale, annoreport was applied to 1500 human gut MAGs from the Unified Human Gastrointestinal Genome catalogue (UHGG v2.0.2; Almeida *et al.* 2021) as a stress test. This dataset was selected for its size and public availability rather than for direct ecological comparison with the Antarctic soil dataset, and the reported runtimes should not be interpreted as a controlled scaling benchmark between the two environments. annoreport was run in GFF-only mode, processing all 1500 MAGs in 232.6 ± 5.1 s with UniProt annotation and 167.0 ± 0.7 s without, with a peak memory usage of 71.9 ± 0.3 MB and 70.3 ± 0.2 MB, respectively.

## 6 Conclusion

annoreport addresses a practical gap in MAG-based metagenomic workflows by providing a lightweight, dependency-free tool for summarizing and visualizing gene annotation results across all bins from Prokka or Bakta output. Beyond simplifying annotation review, annoreport provides a framework for interpreting emergent functional patterns across microbial communities, enabling researchers to move from genome-centric annotation outputs toward ecosystem-scale inference of metabolic and ecological structure. By aggregating gene product frequencies, functional clustering via UniProt annotation, hypothetical protein rates, and feature type distributions into a single interactive HTML report, annoreport enables rapid interpretation of annotation results without requiring additional databases, compute resources, or programming expertise. This capability is particularly relevant for ecosystem-scale and trait-based microbial ecology studies, where interpretation increasingly depends on identifying emergent functional patterns across entire microbial communities rather than within individual genomes alone. The tool is available under an MIT license at https://github.com/keplerridge/annoreport, via Bioconda and PyPI.

Current limitations include reliance on the UniProt Swiss-Prot reviewed database only for functional annotation, which may miss novel or poorly characterized gene products, a consideration that is particularly relevant for environmental metagenomes such as those from extreme environments. COG category reporting is also primarily supported for Prokka output, as Bakta does not natively assign COG categories. The current priority-ordered keyword matching scheme assigns genes to the highest-priority category with any keyword match, regardless of the number of matching keywords. A gene with a single keyword match in DNA Metabolism will be assigned there even if it matches multiple keywords in a lower-priority category. Future development will explore frequency-based assignment, where genes are assigned to the category with the most keyword matches and priority order is used only to break ties, which may improve biological accuracy for multifunctional genes. Future development will focus on multi-sample comparison functionality, pathway-level summaries, expanded database support to improve coverage of novel microbial diversity, and compatibility with additional annotation tools beyond Prokka and Bakta to broaden accessibility across diverse metagenomic workflows.

## Data Availability

The raw metagenomic reads used in this study are available at NCBI BioProject under accession PRJNA1251696 (Thompson *et al.* 2025). The 1500 human gut MAGs used for large-scale benchmarking were sourced from the Unified Human Gastrointestinal Genome catalog v2.0.2 (UHGG; Almeida *et al.* 2021), available at https://www.ebi.ac.uk/metagenomics/genome-catalogues/human-gut-v2-0. The list of specific genomes used for benchmarking is provided in the GitHub repository. The annoreport source code is available at https://github.com/keplerridge/annoreport under an MIT license, via Bioconda and PyPI, and as a reproducible compute capsule on Code Ocean (Ridge and Adams 2026) at https://doi.org/10.24433/CO.0062762.v2.
